# Age at menarche and blood pressure in late adolescence: the role of body adiposity in a Brazilian birth cohort

**DOI:** 10.1590/1980-549720260038

**Published:** 2026-07-31

**Authors:** Juliana Jansen Santos, Janielle Ferreira de Brito Lima, Maria Luziene de Sousa Gomes, Kayo Elmano Costa da Ponte Galvão, Dannyel Rogger Almeida Teixeira, Lua Beatriz Nascimento Soares, Rita da Graça Carvalhal Frazão Corrêa, Rosângela Fernandes Lucena Batista

**Affiliations:** I Universidade Federal do Maranhão, Postgraduate Program in Nursing, Center for Biological and Health Sciences - São Luís (MA), Brazil.; II Universidade Federal do Maranhão, Nursing Course Coordination, Center for Biological and Health Sciences - São Luís (MA), Brazil.; III Universidade Federal do Maranhão, Postgraduate Program in Public Health, Center for Biological and Health Sciences - São Luís (MA), Brazil.; IV Universidade Federal do Maranhão, Nursing Course, Center for Biological and Health Sciences - São Luís (MA), Brazil.; V Universidade Federal do Maranhão, University Hospital - São Luís (MA), Brazil.

**Keywords:** Adolescent health, Menarche, Adiposity, Blood pressure, Saúde do adolescente, Menarca, Adiposidade, Pressão sanguínea

## Abstract

**Objective:**

To investigate the association between age at menarche and blood pressure in adolescence, and to examine the role of body adiposity in this relationship.

**Methods:**

This was a cross-sectional analysis nested within a population-based birth cohort conducted in São Luís, Maranhão, Brazil. The study included 885 female adolescents aged 18-19 years. Age at menarche was self-reported. Systolic and diastolic blood pressure were measured using a validated digital device, and body adiposity was assessed by air displacement plethysmography. Pearson correlation coefficients were estimated, and linear regression models were used to evaluate the association between age at menarche and blood pressure, with sequential adjustment for socioeconomic, behavioral, family, and perinatal factors, followed by additional adjustment for body adiposity.

**Results:**

Age at menarche showed weak inverse correlations with systolic and diastolic blood pressure and a negative correlation with body adiposity. Body adiposity, in turn, was positively correlated with both systolic and diastolic blood pressure. In adjusted models, age at menarche remained significantly associated with blood pressure after controlling for socioeconomic, behavioral, family, and perinatal factors; however, these associations were attenuated and lost statistical significance after additional adjustment for body adiposity.

**Conclusion:**

The association between age at menarche and blood pressure in adolescence appears to be largely influenced by body adiposity, highlighting the importance of considering body composition when evaluating cardiovascular risk related to pubertal timing.

## RESUMO

**Objetivo:**

Investigar a associação entre a idade à menarca e a pressão arterial na adolescência e examinar o papel da adiposidade corporal nessa relação.

**Métodos:**

Trata-se de uma análise transversal aninhada a uma coorte de nascimento de base populacional conduzida em São Luís, Maranhão, Brasil. O estudo incluiu 885 adolescentes do sexo feminino, com idades entre 18 e 19 anos. A idade à menarca foi autorreferida. A pressão arterial sistólica e diastólica foi aferida por dispositivo digital validado, e a adiposidade corporal foi avaliada por pletismografia por deslocamento de ar. Foram estimados coeficientes de correlação de Pearson e utilizados modelos de regressão linear para avaliar a associação entre idade à menarca e pressão arterial, com ajustes sequenciais para fatores socioeconômicos, comportamentais, familiares e perinatais, seguidos de ajuste adicional para adiposidade corporal.

**Resultados:**

A idade à menarca apresentou correlações inversas fracas com a pressão arterial sistólica e diastólica, além de correlação negativa com a adiposidade corporal. A adiposidade corporal, por sua vez, apresentou correlação positiva com ambas as medidas de pressão arterial. Nos modelos ajustados, a idade à menarca manteve associação estatisticamente significativa com a pressão arterial após controle para fatores socioeconômicos, comportamentais, familiares e perinatais; entretanto, essas associações foram atenuadas e perderam significância estatística após ajuste adicional para adiposidade corporal.

**Conclusão:**

A associação entre a idade à menarca e a pressão arterial na adolescência parece ser amplamente influenciada pela adiposidade corporal, destacando a importância de considerar a composição corporal na avaliação do risco cardiovascular relacionado ao tempo puberal.

## INTRODUCTION

Adolescence is a critical period, marked by profound biological, psychological, and social changes that may influence health trajectories into adulthood. According to the World Health Organization, adolescence encompasses individuals aged ten to 19 years and, in females, is characterized by the occurrence of menarche, a key biological event that reflects maturation of the hypothalamic-pituitary-gonadal axis^1^. Over recent decades, a secular trend toward earlier age at menarche has been documented worldwide, including in Brazil, across diverse socioeconomic and cultural contexts^2^ ^,^ ^3^ ^,^ ^4^ ^,^ ^5^.

Age at menarche is determined by a complex interplay of genetic, environmental, socioeconomic, and behavioral factors. Living conditions, dietary patterns, physical activity, body composition, parental education, and household income have all been shown to influence the timing of pubertal onset in girls^2^ ^,^ ^6^ ^,^ ^7^ ^,^ ^8^. Among these determinants, body adiposity has emerged as a key factor, as greater fat mass is associated with earlier reproductive axis activation and anticipation of menarche, suggesting that age at menarche may reflect underlying trajectories of adiposity established earlier.

Earlier age at menarche has been consistently associated with adverse cardiometabolic outcomes later in life, including elevated blood pressure, dyslipidemia, insulin resistance, and increased risk of cardiovascular disease^9^ ^,^ ^10^ ^,^ ^11^ ^,^ ^12^ ^,^ ^13^. Cardiovascular diseases remain a leading cause of preventable morbidity and mortality worldwide, and identifying early-life factors associated with blood pressure elevation is essential for prevention strategies from a life-course perspective^14^.

Body adiposity appears to play a substantial role in the relationship between pubertal timing and blood pressure. Excess body fat during childhood and adolescence has been associated both with earlier pubertal onset and with higher blood pressure levels^11^ ^,^ ^15^ ^,^ ^16^. Moreover, longitudinal studies and meta-analyses indicate that a considerable proportion of the association between age at menarche and blood pressure may be explained by adiposity-related mechanisms^17^ ^,^ ^18^ ^,^ ^19^ ^,^ ^20^.

Despite the growing body of evidence, a key conceptual and analytical issue remains unresolved: whether the association between age at menarche and blood pressure reflects a direct relationship or is largely explained by body adiposity^19^ ^,^ ^21^ ^,^ ^22^. Although several studies have adjusted for anthropometric indicators, many have relied on indirect measures such as body mass index (BMI), which may not adequately capture body fat distribution and composition. Consequently, the extent to which adiposity explains or underlies this association remains uncertain, particularly in studies using more accurate measures of body composition and conducted in low- and middle-income settings.

In this context, adolescence represents a critical window for the early identification of cardiovascular risk factors and for the implementation of preventive strategies capable of modifying long-term health trajectories^23^ ^,^ ^24^. Population-based cohort studies incorporating accurate measures of body composition are therefore essential to clarify the pathways linking pubertal timing, adiposity, and blood pressure during this stage of the life course.

In this study, we conceptualize body adiposity as a variable potentially situated along the pathway linking age at menarche and blood pressure, consistent with its role as a plausible intermediate factor. At the same time, we acknowledge that, in observational analyses based on cross-sectional data, the distinction between mediation and confounding may be limited. Therefore, we adopted a sequential modeling approach to examine how the inclusion of adiposity influences the magnitude of the association between age at menarche and blood pressure.

In this context, the present study aimed to investigate the association between age at menarche and blood pressure among female adolescents participating in a Brazilian population-based birth cohort, while examining the role of body adiposity in this relationship using a precise measure of body composition obtained by air displacement plethysmography.

## METHODS

### Study design and population

This is a cross-sectional analysis nested within a population-based birth cohort in São Luís, Maranhão, Brazil, with follow-up of participants from birth through late adolescence (18-19 years of age). The cohort is part of the RPS Cohort Consortium, a multicenter research project entitled “Determinants across the life course of obesity, precursors of chronic diseases, human capital, and mental health”, developed by the Federal University of Maranhão (UFMA), the Ribeirão Preto Medical School of the University of São Paulo (USP), and the Federal University of Pelotas (UFPel)^25^.

The São Luís birth cohort was established between March 1997 and February 1998 and included 2,443 live births, corresponding to 96.3% of all deliveries that occurred in the ten public and private maternity hospitals in the municipality during that period. A systematic sampling design was adopted, with proportional stratification according to the number of deliveries in each maternity hospital, selecting one out of every seven births. Multiple births, stillbirths, and twin pregnancies were excluded^25^.

Participants were prospectively followed at three time points across the life course: at birth (baseline), during childhood (7-9 years of age), and in late adolescence (18-19 years of age). No additional large-scale follow-ups were conducted between childhood and adolescence^25^.

### Follow-up procedures and participant identification

For the adolescent follow-up, individuals assessed at birth were traced using multiple strategies, including records from the four Military Enlistment Boards in the municipality, the 2014 School Census, and university enrollment databases. Identified participants were invited to attend the assessment conducted between January and December 2016, when they were 18 or 19 years old^25^.

Through this process, 687 adolescents from the original cohort were located and invited to participate. To mitigate cumulative losses during follow-up (resulting from changes in contact information, migration, or deaths), the cohort was expanded by including 1,828 adolescents born in São Luís in the same period (1997), randomly selected from the Brazilian Live Birth Information System (SINASC) and identified through school and university records, according to procedures previously described by the RPS consortium. In total, 2,515 adolescents aged 18-19 years participated in this follow-up, of whom 1,319 were female.

### Study sample

The analytical sample of the present study consisted of female adolescents aged 18-19 years who participated in the third follow-up of the cohort. Among the 1,319 female participants, those with missing information on age at menarche, blood pressure measurements, or covariates included in the analytical models were excluded. In addition, adolescents who reported the use of antihypertensive medications at the time of assessment were excluded.

After applying these criteria, the final analytical sample comprised 885 adolescents.

### Exposure variable

The exposure variable was age at menarche, obtained through the question: “How old were you when you menstruated for the first time?”, included in the reproductive health questionnaire administered to the adolescents. For descriptive purposes only, a categorical variable “early menarche” was created, defined as menarche occurring at 11 years of age or younger, according to criteria adopted in national studies of Brazilian adolescents^26^. In correlation and regression analyses, age at menarche was treated as a continuous variable.

### Outcome variable

The outcomes were systolic blood pressure (SBP) and diastolic blood pressure (DBP), measured using a validated digital sphygmomanometer (Omron HEM-742INT), with cuffs appropriate to arm circumference. Measurements were performed by a trained evaluator after at least five minutes of rest, with participants seated and the left arm supported at heart level. Three consecutive measurements were obtained, and the mean of the last two was used, in accordance with recommendations of the Brazilian Society of Pediatrics^15^.

For descriptive purposes, blood pressure was classified as normal (SBP <120 mmHg and DBP <80 mmHg), prehypertension (SBP 120-139 mmHg and/or DBP 80-89 mmHg), and hypertension (SBP ≥140 mmHg and/or DBP ≥90 mmHg), according to the Brazilian Guidelines of Hypertension^27^. For statistical analyses, SBP and DBP were treated as continuous variables.

### Covariates

Covariates were selected based on prior evidence and organized into sociodemographic, behavioral, family, and perinatal domains. These included age (18 or 19 years); self-reported skin color (White; Black; Brown/mixed); economic class according to the Brazilian Economic Classification Criterion (A/B; C; D/E); monthly household income; adolescent’s educational level; head of household’s educational level; parental separation or divorce (yes/no); adolescent smoking (yes/no); alcohol consumption in the past 12 months (yes/no); maternal smoking during pregnancy (yes/no); and birth weight (grams).

Exposure to stressful life events was assessed by combining responses to the questions: “Have you ever felt afraid or unsafe in your neighborhood?” and “Have you ever been robbed?”, and was classified as present or absent.

Leisure-time physical activity was assessed using the Self-Administered Physical Activity Checklist (SAPAC), a validated instrument for adolescents that estimates physical and sedentary activities over the previous 24 hours. Based on estimated energy expenditure in MET-min/day, adolescents were classified as sedentary or as having low, moderate, or high levels of physical activity, according to criteria proposed by Sallis et al.^28^

BMI was calculated as weight (kg) divided by height squared (m^2^). Weight and height were measured using standardized procedures. BMI was classified according to World Health Organization criteria for adults, as underweight (<18.5 kg/m^2^), normal weight (18.5-24.9 kg/m^2^), overweight (25.0-29.9 kg/m^2^), and obesity (≥30.0 kg/m^2^)^29^. For descriptive analyses, BMI was categorized accordingly; it was not included in the main regression models due to its conceptual overlap with body fat percentage.

### Assessment of body adiposity

Body adiposity was assessed using air displacement plethysmography with the Bod Pod® Gold Standard device (COSMED), a method considered highly accurate for estimating body fat percentage. Body fat percentage was classified according to criteria proposed by Williams et al.^30^, with values ≥30% considered elevated for females.

For statistical analyses, body fat percentage was treated as a continuous variable and was included in the regression models as the main indicator of adiposity.

### Statistical analysis

Statistical analyses were performed using Stata® software, version 14.0 (StataCorp, College Station, Texas, USA). Initially, descriptive analyses were conducted, with categorical variables presented as absolute and relative frequencies and continuous variables as means and standard deviations. Differences in the distribution of body mass index categories and high body fat percentage according to early menarche were assessed using the Pearson ꭓ^2^ test. The distribution of continuous variables was assessed using histograms as well as skewness and kurtosis measures, with no relevant deviations from normality identified that would justify data transformation.

Pearson correlation coefficients were used to examine correlations between age at menarche, body adiposity, and systolic and diastolic blood pressure. The magnitude of correlations was interpreted according to criteria proposed by Cohen^31^.

**Figure 1.**
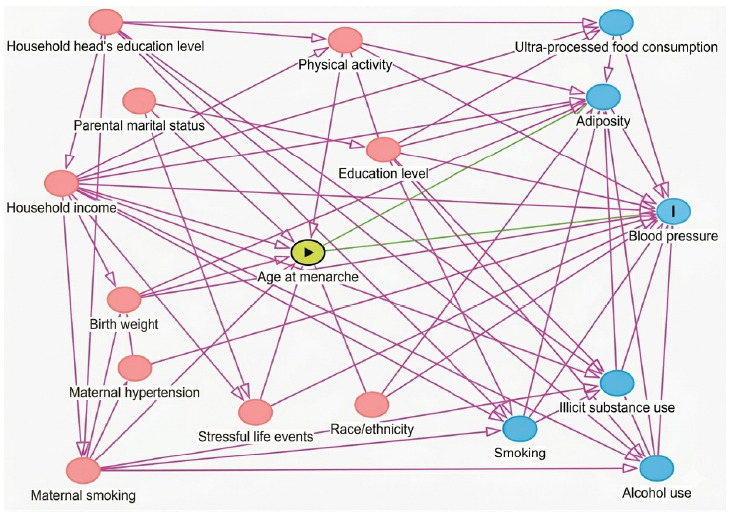
Directed acyclic graph illustrating the relationship between age at menarche, body adiposity, and blood pressure in female adolescents.

Source: The authors.

Based on evidence from the literature and a priori theoretical assumptions, a directed acyclic graph (DAG) was constructed using DAGitty® software, version 3.0, to represent the hypothesized relationships among age at menarche, body adiposity, blood pressure, and potential confounders. The DAG was used to identify the minimal sufficient adjustment set required to minimize confounding in the association between age at menarche and blood pressure (Figure 1).

Linear regression models were used to assess the association between age at menarche and systolic and diastolic blood pressure in adolescence. First, crude associations were estimated using simple linear regression (Model 1). Next, a multiple linear regression model was constructed (Model 2), adjusted for the minimal sufficient set of confounders identified by the DAG, namely: household income, leisure-time physical activity, exposure to stressful life events, birth weight, parental separation or divorce, and maternal smoking during pregnancy.

In the final model (Model 3), body adiposity was additionally included to explore its potential role along the pathway linking age at menarche and blood pressure, given its plausibility as an intermediate variable. This approach allows evaluation of the extent to which the observed association is attenuated after inclusion of adiposity, without implying formal mediation analysis.

Results are presented as beta coefficients (β) with corresponding 95% confidence intervals (95%CI) and p-values. A two-sided significance level of 5% was adopted for all analyses.

### Ethical considerations

The study was conducted in accordance with the principles of the Declaration of Helsinki and complied with Brazilian ethical guidelines for research involving human subjects, as established in Resolution No. 466/2012 of the National Health Council^32^. The research protocol was approved by the Research Ethics Committee of the University Hospital of the Federal University of Maranhão (approval number 1.302.489, October 29, 2015). All participants provided written informed consent prior to participation.

## Data availability statement:

The data that support the findings of this study are not publicly available due to ethical and confidentiality restrictions related to participant privacy. Data may be made available from the corresponding author upon reasonable request and subject to approval by the study investigators and the relevant ethics committee.

## RESULTS

A total of 885 female adolescents aged 18-19 years were included in the analyses. Most participants were 18 years old (66.2%) and self-identified as Brown/mixed (61.3%). The majority reported 9-11 years of schooling (89.4%), and just over half of household heads had completed secondary education (52.8%). Nearly half of the adolescents had parents who were separated or divorced (46.6%). Most families reported a monthly income of one minimum wage (34.6%) and belonged to economic class C (48.5%) (Table 1).

**Table 1.** Sociodemographic and family characteristics of female adolescents from the RPS birth cohort in São Luís (MA), Brazil, 2016.

Variables	n (%)
Age (years)	
18	586 (66.2)
19	299 (33.8)
Skin color*	
White	196 (22.2)
Black	145 (16.5)
Brown/mixed	540 (61.3)
Adolescent’s education (years of schooling)	
0-8	30 (3.4)
9-11	788 (89.4)
≥12	63 (7.2)
Household head’s education	
No formal education	8 (1.0)
Elementary school	222 (27.1)
High school	433 (52.8)
Incomplete higher education	30 (3.6)
Complete higher education	127 (15.5)
Parents separated/divorced	
No	473 (53.4)
Yes	412 (46.6)
Family income (minimum wages)	
<1	121 (13.7)
1	306 (34.6)
2	199 (22.5)
3	94 (10.6)
≥4	165 (18.6)
Economic class*	
A/B	230 (28.3)
C	394 (48.5)
D/E	188 (23.2)

*Values are presented as n (%) unless otherwise indicated. Percentages may not total 100% due to missing data.

Source: The authors.

Regarding lifestyle, perinatal, and health-related characteristics, exposure to stressful events was highly prevalent (91.2%). Most adolescents were classified as sedentary during leisure time (62.3%). Smoking was reported by a small proportion of participants (5.4%), while alcohol consumption in the previous 12 months was reported by 53.5%. Maternal smoking during pregnancy was reported by 3.7% of mothers. Mean birth weight was 3,146.4 g (±603.1 g) (Table 2).

**Table 2.** Lifestyle, perinatal, and health-related characteristics of female adolescents from the RPS birth cohort in São Luís (MA), Brazil, 2016.

Variables	n (%)
Stressful events	
No	78 (8.8)
Yes	807 (91.2)
Leisure-time physical activity	
Sedentary	551 (62.3)
Low	111 (12.5)
Moderate	146 (16.5)
High	77 (8.7)
Maternal smoking during pregnancy	
No	852 (96.3)
Yes	33 (3.7)
Adolescent smoking	
No	837 (94.6)
Yes	48 (5.4)
Adolescent alcohol consumption (last 12 months)*	
No	410 (46.5)
Yes	471 (53.5)
Early menarche (years)	
>11	594 (67.1)
≤11	291 (32.9)
Blood pressure levels in adolescents	
Normal blood pressure	774 (87.5)
Prehypertension	103 (11.6)
Hypertension	8 (0.9)
High body fat percentage	
No	513 (58)
Yes (≥30%)	372 (42)
Body mass index	
Underweight	165 (18.6)
Normal weight	542 (61.3)
Overweight	140 (15.8)
Obesity	38 (4.3)
**Variables**	**Mean (±SD)**
Age at menarche (years)	12.2 (1.4)
Systolic blood pressure (mmHg)	107.2 (9.4)
Diastolic blood pressure (mmHg)	69.8 (6.9)
Birth weight (grams)	3,146.4 (603.1)
Body fat percentage	28.3 (8.4)
Body mass index (kg/m^2^)	22.1 (4.2)

*Values are presented as n (%) or mean±standard deviation, as appropriate. Percentages may not total 100% due to missing data.

Source: The authors.

The mean age at menarche was 12.2 years (±1.4), and the prevalence of early menarche (≤11 years) was 32.9%. Hypertension prevalence was low (1.2%). Mean systolic and diastolic blood pressure values were 107.2 mmHg (±9.4) and 69.8 mmHg (±6.9), respectively. Elevated body fat percentage (≥30%) was observed in 42.0% of adolescents, with a mean body fat percentage of 28.3% (±8.4) (Table 2).

Table 3 shows the distribution of BMI and high body fat percentage according to early menarche. Adolescents with early menarche (≤11 years) presented a higher proportion of overweight and obesity compared to those with later menarche (27.1 vs. 16.7%), as well as a higher prevalence of elevated body fat percentage (48.5 vs. 38.9%) (p<0.01 for both comparisons).

**Table 3.** Distribution of body mass index and high body fat percentage according to age at menarche in female adolescents from the RPS birth cohort in São Luís (MA), Brazil, 2016.

Variables	Early menarche		p-value*
	>11 years n (%)	≤11 years n (%)	
Body mass index			
Underweight	118 (19.9)	47 (16.2)	0.003
Normal weight	377 (63.5)	165 (56.7)	
Overweight	80 (13.5)	60 (20.6)	
Obesity	19 (3.2)	19 (6.5)	
High body fat percentage			
No	363 (61.1)	150 (51.5)	0.007
Yes (≥30%)	231 (38.9)	141 (48.5)	
Total	594 (100)	291 (100)	

*Pearson ꭓ^2^.

Source: The authors.

**Figure 2.**
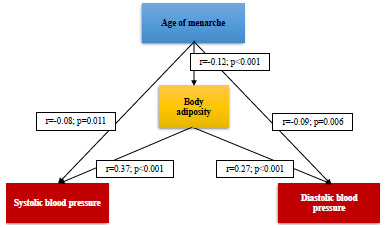
Pearson correlation coefficients between age at menarche, body adiposity and blood pressure among female adolescents from the RPS birth cohort in São Luís. São Luís (MA), Brazil, 2016.

Source: The authors.

Pearson correlation analyses showed a weak inverse correlation between age at menarche and body adiposity (r=-0.12; p<0.001). Body adiposity was moderately and positively correlated with systolic blood pressure (r=0.37; p<0.001) and weakly correlated with diastolic blood pressure (r=0.27; p<0.001). Weak inverse correlations were also observed between age at menarche and systolic (r=-0.09; p=0.006) and diastolic blood pressure (r=-0.08; p=0.011) (Figure 2).

Table 4 presents the results of linear regression analyses examining the association between age at menarche and blood pressure. In the unadjusted model (Model 1), each one-year increase in age at menarche was associated with lower systolic blood pressure (β=-0.56; 95%CI -0.98 to -0.13) and diastolic blood pressure (β=-0.44; 95%CI -0.75 to -0.12). These associations remained after adjustment for household income, leisure-time physical activity, exposure to stressful events, birth weight, parental separation or divorce, and maternal smoking during pregnancy (Model 2).

In the final model, which additionally included body adiposity (Model 3), the associations between age at menarche and both systolic (p=0.147) and diastolic blood pressure (p=0.072) were attenuated and lost statistical significance after adjustment for body adiposity (Table 4).

**Table 4.** Linear regression models for the association between age at menarche and systolic and diastolic blood pressure among female adolescents from the RPS birth cohort in São Luís (MA), Brazil, 2016.

	Blood pressure	
	Systolic	Diastolic
Model 1		
β	-0.56	-0.44
95%CI	-0.98; -0.13	-0.75; -0.12
p-value	0.011	0.006
Model 2		
β	-0.58	-0.43
95%CI	-1.01; -0.16	-0.74; -0.11
p-value	0.008	0.007
Model 3		
β	-0.30	-0.28
95%CI	-0.70; 0.10	-0.58; 0.25
p-value	0.147	0.072

β: regression coefficient; CI: confidence interval.

Model 1: unadjusted.

Model 2: adjusted for family income, leisure-time physical activity, exposure to stressful events, birth weight, parental separation/divorce, and maternal smoking during pregnancy.

Model 3: additionally adjusted for body fat percentage (air displacement plethysmography).

Source: The authors.

## DISCUSSION

In this population-based study of female adolescents from a Brazilian birth cohort, age at menarche was inversely associated with systolic and diastolic blood pressure in crude and adjusted models; however, these associations were attenuated and no longer statistically significant after inclusion of body adiposity. These findings indicate that body composition plays a central role in the relationship between pubertal timing and blood pressure during late adolescence and are consistent with epidemiological evidence suggesting that adiposity represents an important intermediate mechanism linking early menarche to cardiometabolic risk across the life course^9^ ^,^ ^20^.

The inverse association observed between age at menarche and body adiposity indicates that earlier menarche tends to occur among adolescents with higher body fat percentage. Similar patterns have been reported in national and international studies, which demonstrate that greater energy availability and adipose tissue accumulation favor earlier activation of the hypothalamic-pituitary-gonadal axis, leading to earlier pubertal onset^10^ ^,^ ^11^. In this context, age at menarche may act as a biological marker of underlying adiposity trajectories established earlier in life^2^ ^,^ ^3^ ^,^ ^5^ ^,^ ^6^ ^,^ ^7^ ^,^ ^8^.

The attenuation of associations after inclusion of body adiposity in the regression models suggests that this variable may play a relevant role in the relationship between age at menarche and blood pressure. However, this interpretation should be made with caution, as the inclusion of a potentially intermediate variable in regression models may reduce the magnitude of associations without necessarily representing adjustment for confounding. Thus, the findings are consistent with the hypothesis that body adiposity lies along the pathway linking pubertal timing to blood pressure, although formal mediation cannot be established.

The findings of this study should be interpreted considering the role of age at menarche as a biological marker of underlying processes rather than a direct causal factor for blood pressure alterations. Earlier menarche is strongly associated with trajectories of adiposity and nutritional status throughout childhood and adolescence^5^ ^,^ ^9^ ^,^ ^18^, which, in turn, influence blood pressure levels^19^ ^,^ ^20^. Therefore, age at menarche may reflect early metabolic exposures shared with body adiposity, functioning as an indirect indicator within a broader causal framework.

Regarding blood pressure, the positive association between body adiposity and both systolic and diastolic blood pressure observed in this study is consistent with previous literature. This relationship may be explained by obesity-related pathophysiological mechanisms, including increased peripheral vascular resistance, sympathetic nervous system activation, chronic low-grade inflammation, and metabolic alterations^33^ ^,^ ^34^. Evidence from studies involving adolescents and young adults indicates that excess body fat constitutes an important determinant of elevated blood pressure early in the life course.

The persistence of the association between age at menarche and blood pressure after adjustment for socioeconomic, behavioral, family, and perinatal factors suggests that these variables do not fully explain the observed relationship. However, the loss of statistical significance after inclusion of body adiposity reinforces that this association is not independent of body composition. This finding is in line with observational studies and meta-analyses indicating that adiposity substantially accounts for the relationship between pubertal timing and cardiometabolic outcomes^18^ ^,^ ^19^.

Differences between studies reporting independent associations may be partly explained by methodological aspects. In the present study, body adiposity was assessed using air displacement plethysmography, a method considered more accurate for estimating body fat percentage than indirect anthropometric measures such as body mass index^30^. The use of a precise measure of adiposity may have allowed a clearer identification of its role in this association.

From a methodological perspective, the use of a directed acyclic graph to define the minimal sufficient adjustment set strengthens the internal validity of the study by reducing the risk of inappropriate adjustment for confounding. Although a formal mediation analysis was not performed, the sequential modeling strategy adopted provides evidence consistent with a potential intermediate role of body adiposity, as suggested in previous epidemiological ­studies^19^ ^,^ ^20^.

The interpretation of these findings should consider the temporal nature of the variables analyzed. Although age at menarche represents a prior event, both body adiposity and blood pressure were measured at the same time point, characterizing a cross-sectional analysis. This limits causal inference regarding the direction of the associations, and bidirectional or shared developmental pathways cannot be ruled out. Longitudinal studies with repeated measures of adiposity are needed to better clarify these relationships.

Some limitations should be acknowledged. Age at menarche was self-reported and may be subject to recall bias, although previous studies suggest acceptable reliability. In addition, pre-menarche adiposity measures were not available, limiting the assessment of temporality. Nevertheless, adiposity measured in late adolescence likely reflects earlier body composition trajectories. Strengths of this study include the use of a population-based birth cohort, the application of a precise method for adiposity assessment, and an analytical approach guided by a causal framework.

The low prevalence of elevated blood pressure in the study population should also be considered. In settings with low outcome variability, statistical power to detect associations, particularly of small magnitude, is reduced, which may lead to attenuated estimates and wider confidence intervals. Therefore, the absence of statistically significant associations in adjusted models should be interpreted with caution.

Additionally, potential selection bias due to losses to follow-up and sample recomposition cannot be ruled out. Differences between included and non-included participants, particularly in socioeconomic or behavioral characteristics, may have influenced the estimates.

In summary, the findings indicate that the association between age at menarche and blood pressure in late adolescence is largely explained by body adiposity. These results contribute to a more precise understanding of early-life mechanisms linking pubertal timing to cardiovascular risk and reinforce the importance of addressing excess adiposity during adolescence as a strategy to reduce future cardiometabolic risk.
